# Lightweight audio-visual sentiment analysis with dynamic mouth-state modeling and multimodal LLM benchmarking

**DOI:** 10.3389/frai.2026.1855069

**Published:** 2026-10-29

**Authors:** Atitaya Yakaew, Teeradaj Racharak, Matthew N. Dailey, Watanee Jearanaiwongkul

**Affiliations:** 1 Faculty of Advanced Science and Technology (FAST), Asian Institute of Technology (AIT), Pathum Thani, Thailand; 2 Advanced Institute of So-Go-Chi (Convergence Knowledge) Informatics, Tohoku University, Sendai, Japan

**Keywords:** dynamic mouth state analysis, multimodal large language models, multimodal sentiment analysis, RAVDESS, sentiment classification, temporal feature fusion

## Abstract

Sentiment analysis from video streams requires effective modeling of both temporal dynamics and multimodal cues. This paper presents a lightweight multimodal CNN-LSTM framework, designed for low-latency operation on live video streams, that classifies individuals' affective states as negative, neutral, or positive by jointly exploiting audio and visual information. The proposed system consists of four lightweight deep neural network models that fuse sequences of audio and visual features into a unified temporal stream, enabling the accumulation of sentiment evidence over time for robust prediction. To further enhance discriminative power, dynamic mouth state classification is incorporated, allowing the model to better capture subtle visual cues related to speech and sentiment. Experiments conducted on the Ryerson Audio-Visual Database of Emotional Speech (RAVDESS) demonstrate that the proposed approach achieves a test accuracy of 84.89% on the three-class sentiment recognition task, competitive with substantially larger audio-visual architectures, while using an order of magnitude fewer parameters and maintaining low-latency operation. In addition, we provide a comprehensive comparison with multiple multimodal large language models (LLMs), evaluated under diverse input configurations and prompting strategies. The results offer practical insights into the strengths and limitations of contemporary LLMs for audio-visual sentiment analysis and highlight the continued effectiveness of compact, task-specific neural architectures for streaming audio-visual input.

## 1 Introduction

In competitive business environments, customer feedback plays a critical role in shaping strategic decision making and service optimization. Conventional feedback collection methods, such as questionnaires and focus groups, are easy to deploy but often suffer from low participation rates and limited temporal coverage. Thus, they fail to capture customers' spontaneous reactions during real-world interactions. An alternative approach is pervasive, real-time, and passive feedback collection during the customer experience itself. Human sentiment can be expressed through multiple modalities, including text, speech, and facial expressions, and recent advances in data processing and deep learning have enabled the automated analysis of such multimodal signals at scale.

Among these modalities, video provides a particularly rich source of affective information. Modern environments are increasingly equipped with intelligent video capture devices, especially in retail and service settings where cameras are deployed for security and operational purposes. The continuous streams generated by these systems represent a largely under-utilized resource for understanding customer sentiment, as they naturally capture both facial expressions and accompanying speech without requiring explicit user interaction.

Classifiers for emotion facial expression using deep learning techniques and feature engineering have been under development since the early 1990's. Cottrell and Metcalfe (1990) used back propagation networks for face recognition, emotion state classification, and identification of gender. Dailey et al. (2002) later applied Gabor jets, PCA, and multi-layer perceptrons to the task of facial expression recognition in single images. Bartlett et al. (2003) integrated automatic face tracking and expression analysis into automatic animated tutoring systems, and their work has also been applied to customer feedback. Bartlett et al.'s work was among the first to use data-driven feature selection with Adaboost and SVMs to enhance facial expression classification performance. More recently, as in virtually every visual recognition task, data-driven feature selection via CNNs and CNN-LSTMs (You et al., 2015; Jain et al., 2018) has proven even more successful.

In the text domain, sentiment analysis research has focused on developing representations that capture contextual and sequential information in natural language. Earlier approaches employed n-gram-based features and manually designed linguistic cues (Go et al., 2009; Pak and Paroubek, 2010; Pang et al., 2002; Barbosa and Feng, 2010), while more recent methods leverage distributed word and sentence representations learned by deep neural models such as BERT and related architectures (Sun et al., 2019; González-Carvajal and Garrido-Merchán, 2020). When combined with sequential models, including long short-term memory networks (LSTMs) (Hochreiter and Schmidhuber, 1997) and Transformers (Vaswani et al., 2017), these representations have led to significant gains in sentiment classification performance.

Similarly, in the audio domain, speech emotion recognition has benefited from both handcrafted and learned acoustic features. Commonly used features include pitch-related, energy-based, and spectral descriptors extracted from speech signals. With the adoption of deep learning techniques analogous to those used in text and image analysis, audio-based sentiment and emotion recognition systems have demonstrated strong performance across a range of benchmarks (El Ayadi et al., 2011; Venkataramanan and Rajamohan, 2019).

Despite these advances, much of the existing work has focused on a single modality, treating text, audio, and visual information in isolation. In contrast, video data inherently contain both audio and visual streams, offering a richer and more complementary source of affective information. Recent surveys have highlighted a growing shift from a single modality to multimodal sentiment analysis where audio, visual, and text are jointly leveraged to improve sentiment prediction (Das and Singh, 2023; Singh et al., 2024). Fusing these modalities can exploit complementary information from different modalities, enabling a learning of more robust and temporally consistent representations for emotion and sentiment recognition (Glorot et al., 2011; Jayalekshmi and Mathew, 2017; Zhang et al., 2018; Cai et al., 2025). This multimodal perspective is particularly relevant for applications such as customer satisfaction analysis, where affective cues are expressed through concurrent speech and facial behavior (Seng and Ang, 2017; Rokade and Aruna, 2019; Al-Azani and El-Alfy, 2020). This work demonstrates that jointly modeling audio and visual components of video streams leads to higher sentiment classification accuracy compared with unimodal approaches.

The objective of this work is to enable low-latency sentiment prediction from live video streams through data-driven multimodal feature extraction. The main contributions of this paper are as follows:

1. We propose a multimodal sentiment analysis framework that integrates CNN and LSTM architectures to dynamically fuse audio and visual information from real-time video input, classifying sentiment into three categories: negative, neutral, and positive.
2. Extensive experiments on the Ryerson Audio-Visual Database of Emotional Speech (RAVDESS) (Livingstone and Russo, 2018) demonstrate that the proposed approach achieves accuracy competitive with substantially larger audio-visual architectures on the three-class sentiment recognition task, while using an order of magnitude fewer parameters (20.9M vs. 270M+ in Song et al., 2021), underscoring the efficiency of the proposed design for low-latency deployment.

The proposed multimodal deep learning model operates on sequences of paired face images and corresponding spectrogram representations extracted from video frames. Sentiment predictions are inferred over temporal windows rather than individual frames, allowing the model to accumulate affective evidence over time. Notably, the entire architecture contains only 20,897,598 trainable parameters, making it computationally efficient while still achieving accuracy competitive with substantially larger prior architectures on the RAVDESS three-class sentiment task. Experimental results and comparisons with existing approaches are presented in Sections 3 and 4, followed by conclusions in Section 5.

In addition to conventional deep learning models, recent advances in large language models (LLMs) and vision-language models have raised questions about their applicability to multimodal sentiment analysis tasks. To address this, this paper (Subsection 3.5) also provides a systematic experimental comparison between the proposed CNN-LSTM framework and multiple multimodal LLMs under diverse input configurations and prompting strategies. This comparison offers empirical insights into the strengths and limitations of general-purpose LLMs for audio-visual sentiment recognition and helps clarify the role of lightweight, task-specific architectures in real-time affective computing.

## 2 Sequential audio-visual sentiment analysis for live video streams

Our CNN-LSTM network has two parallel feature extraction states, one for audio features and one for visual features. The model performs multimodal feature fusion over entire videos for sentiment classification (negative, neutral, and positive). The system comprises four modules: (1) face and mouth detection, (2) mouth classification, (3) sequential visual sentiment analysis, and (4) fused sequential audio-visual sentiment classification. A detailed view of the pipeline is given in Figure 1:

1. Video is captured at 35 ms intervals. The face, if detected, is cropped and resized to 96 × 96. We buffer the most recent 10 face images in a track as input for the remaining steps.
2. For each face frame, the system detects mouth landmarks and resizes the mouth region to 28 × 28. The mouth state (closed or open) is predicted using a small CNN (cf. Section 2.2).

a. When the mouth is closed for the entire 10-frame buffer, we focus on the visual data only using a visual CNN and LSTM (cf. Section 2.3).
b. Otherwise (the mouth is open in one or more frames in the buffer), audio features in the form of spectrograms are obtained by applying the short time Fourier transform (STFT) to the audio signal and rendering spectrogram images at size 400 × 400. The series of spectrograms and face images are processed at the same time; the audio CNN-LSTM and visual CNN-LSTM branches are concatenated prior to overall classification. The outputs of the model, beginning with the 10th frame and ending when the face track ends, are averaged (cf. Section 2.5).

3. The average output for the track is used as the sentiment prediction for the entire track.

**Figure 1:**
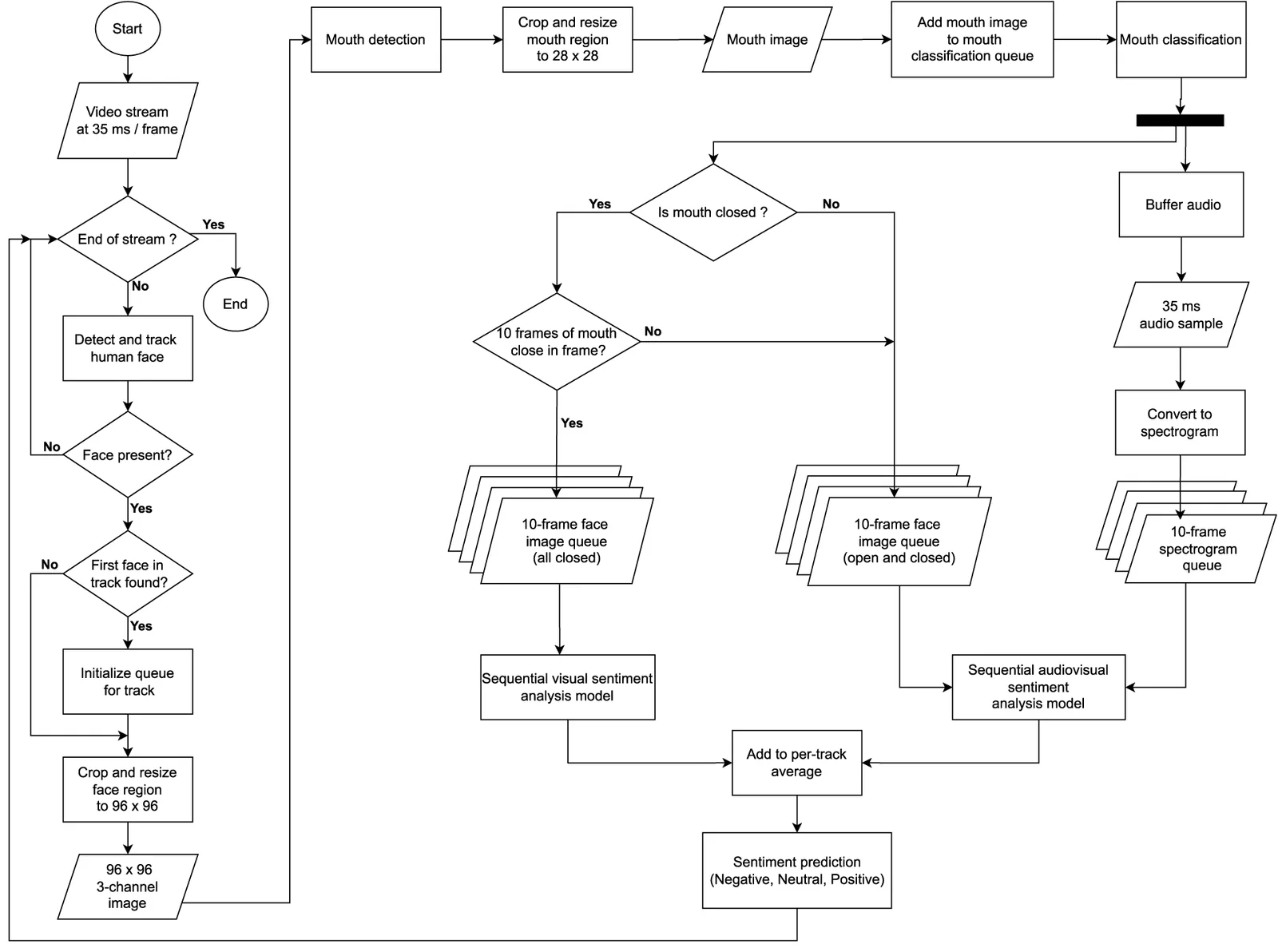
Overall system diagram of the proposed approach.

### 2.1 Face and mouth detection

The system starts when a video stream is available. The video frames are extracted every 35 ms from the input video stream. For face detection, we use the classic histogram of oriented gradient (HOG) detector with a linear SVM. We utilize dlib's facial landmark detector to obtain the locations of 68 fiducial points that map to different components of the face. Based on the feature point locations, each video frame's face and mouth region are cropped as separate images. The mouth region corners are the extrema of dlib landmark indices numbered 49–63. Each face is resized to 96 × 96, and each mouth is resized to 28 × 28.

### 2.2 Mouth classification

The second stage in the flow is mouth classification. The training module takes labeled 28 × 28 × 3 mouth regions as input and produces a mouth classification model. Figure 2 shows the architecture of the mouth classifier model, which is a CNN consisting of five layers. The input mouth image is resized to the fixed size of 28 × 28 × 3 then passed through several convolutional layers. The model architecture is similar to that proposed by Yakaew et al. (2021), with two convolutional layers with ReLU activation functions and max-pooling followed by two fully-connected layers, also with ReLU activations, which are in turn followed by a logistic sigmoid binary output. The output represents the posterior probability that the input represents an “open” mouth, which we threshold at 0.5 to obtain a crisp prediction from the network (Equation 1):


$$\begin{array}{l} a_{t}^{m}\leftarrow {ŷ}_{t}^{m}\geq 0.5. \end{array}$$ (1)

After classifying the mouth as open or closed at time *t*, the frame, the mouth state, and the audio samples, represented by their spectrograms, are buffered. The system monitors the mouth state over a sequence of frames. When the mouth is open in any of the frames in the buffer, both the audio and visual sequences are passed to the sequential audio-visual sentiment analysis model for predicting sentiment. On the other hand, when the mouth classification indicates the mouth is closed for all frames in the buffer, the system passes the visual sequence to the sequential visual sentiment analysis model. We use a buffer length of 10 in our experiments. Examples of mouth regions classified as closed and open with the detected mouth landmarks marked are shown in Figure 3.

**Figure 2:**
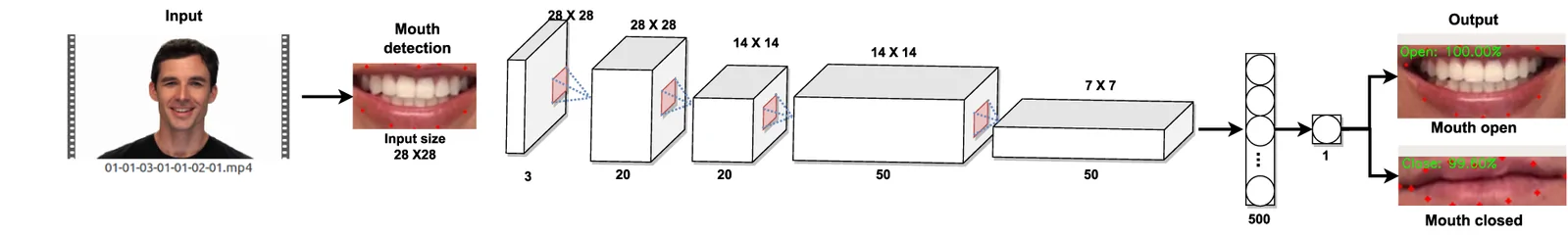
Mouth classification model (input images adapted with permission from The Ryerson Audio-Visual Database of Emotional Speech and Song [RAVDESS], Livingstone and Russo, (2018), https://zenodo.org/records/1188976, licensed under CC BY-NC-SA 4.0).

**Figure 3:**
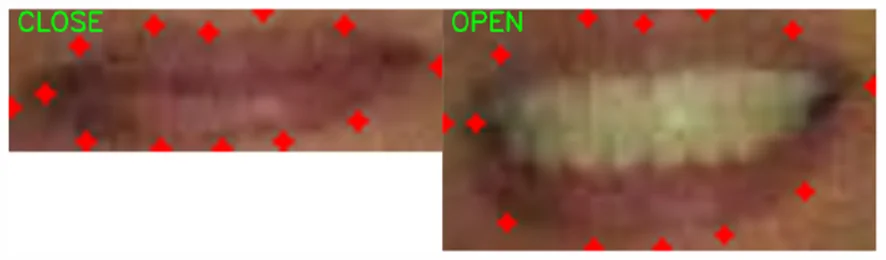
Real-time mouth classification (input images adapted with permission from The Ryerson Audio-Visual Database of Emotional Speech and Song [RAVDESS], Livingstone and Russo, (2018), https://zenodo.org/records/1188976, licensed under CC BY-NC-SA 4.0).

### 2.3 Sequential visual sentiment analysis

When mouth classification reports a closed mouth for the entire buffer, automated sentiment analysis is based on the series of human face images from the video stream. The CNN extracts features from each frame, and the LSTM aggregates the information over time. The outputs of all the hidden states in the LSTM layers are averaged by a mean pooling layer. Finally, a fully-connected layer and a softmax layer are utilized to obtain final classification scores. The three outputs represent a possible negative, neutral, or positive prediction. The visual processing model is shown in Figure 4.

**Figure 4:**
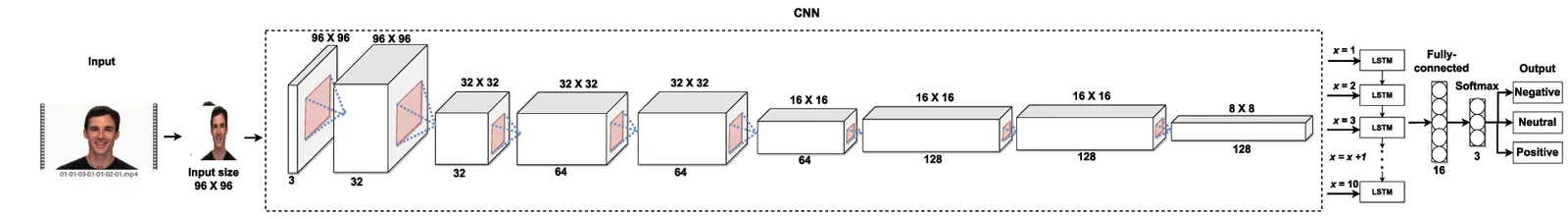
Visual CNN-LSTM model (input image adapted with permission from The Ryerson Audio-Visual Database of Emotional Speech and Song [RAVDESS], Livingstone and Russo, (2018), https://zenodo.org/records/1188976, licensed under CC BY-NC-SA 4.0).

### 2.4 Sequential audio-visual sentiment analysis

When the mouth classifier reports an open mouth for any of the frames in the buffer, the speaker's sentiment is predicted based on both input modalities: the series of audio data (spectrograms) and the series of visual data (face frames). The two types of features are processed concurrently by separate CNN-LSTM models. The visual model, already discussed, is shown in Figure 4, and the audio model is shown in Figure 5. The two models have different sizes and shapes but similar structures, with nine convolutional layers, three pooling layers, one batch normalization layer, one flattening layer, one fully connected layer, one LSTM layer, and one output layer. The convolutional layers use kernel size 3 × 3 and ReLU activation. The max-pooling layers use kernel size 2 × 2 and stride of 2 to reduce feature map dimensionality. We apply 20% dropout to the LSTM layers.

**Figure 5:**
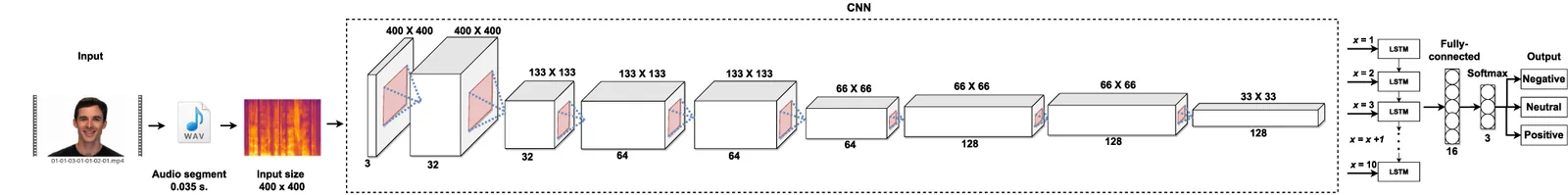
Audio CNN-LSTM model (input image adapted with permission from The Ryerson Audio-Visual Database of Emotional Speech and Song [RAVDESS], Livingstone, and Russo (2018), https://zenodo.org/records/1188976, licensed under CC BY-NC-SA 4.0).

The audio spectrograms indicate power at different audio frequencies over time. The speech signal segments are transformed in real time along with video frames using a short time Fourier transform (STFT) to obtain spectrogram entry *S*_*c*_ (*n, k*), where *n* and *k* are the time frame and frequency indices, respectively, from the audio signal *x*(*n*), which is sampled at 48 kHz. The full audio signal *x*(*n*) is sliced into overlapping segments of length *W*, computed every *h* samples, where *h* is the hop size (the number of samples the analysis window advances between consecutive frames); the overlap between consecutive windows is therefore *W*−*h* samples, or (*W*−*h*)/*W*×100%. We set the STFT window size *W* to 256 samples (5.33 ms) and the hop size *h* to 6 samples (0.125 ms), corresponding to an overlap of 250 samples (97.7%) between consecutive windows. Each segment is multiplied element-wise by a Tukey window *w*(*l*) with shape parameter 0.25, for *l* = −*W*/2, …, *W*/2 − 1, before the summation in Equation 2 is evaluated over this same range, yielding 129 frequency bins. Time is plotted on the long X-axis, and the Y-axis represents frequency (0–24 kHz). The spectrogram elements are rendered on a dB scale (10log_10_ of the power).


$$\begin{array}{l} S_{c}\left(n,k\right)\leftarrow \overset{\frac{W}{2}-1}{\underset{l=-\frac{W}{2}}{\sum }}w\left(l\right)\cdot x\left(l+nh\right)\cdot e^{-2\pi ilk/W} \end{array}$$ (2)

The neural network architecture has two sub-networks: the audio model and the visual model. Both models include two sub-modules: the CNNs for feature extraction and the LSTMs for learning the temporal correlation of sequences. The two inputs (series of faces and series of spectrograms) are passed to the audio model and the visual model, each of which includes as sub-models a CNN and LSTM that run in parallel. Finally, the system fuses the outputs of the audio and visual models by concatenating their representations. The last state is a softmax over the three sentiment classes (negative, neutral, and positive).

### 2.5 Real-time sentiment aggregator

Regardless of which branch is activated for a particular sequences from frame 10 of each track onwards, predictions are averaged, and the analyzer outputs the sentiment class with the highest estimated probability over the track.

## 3 Experiments

### 3.1 Dataset

This study focuses on sentiment recognition using the emotion categories defined in the RAVDESS dataset. The Ryerson audio-video database of emotional speech (RAVDESS) was recorded by Livingstone and Russo (Livingstone and Russo, 2018).^1^ It contains 24 professional actors (12 females and 12 males). The speech is in a neutral North American accent. Speech includes eight categories: neutral, calm, happy, sad, angry, fearful, surprise, and disgust expressions. Not all human emotions provide clear feedback for businesses monitoring customer satisfaction. For example, the emotion of surprise may indicate either positive or negative sentiment depending on the context; disgust and fear are similarly context-dependent. Therefore, we focus on five categories: neutral, calm, happy, sad, and angry. This grouping follows a dimensional (valence-based) view of affect (Russell, 1980), under which calm and neutral occupy a near-zero-valence region and are grouped as “neutral,” happy is positive-valence and retained as “positive,” and angry and sad are both negative-valence and grouped as “negative,” while surprise, disgust, and fear were excluded because their valence is not consistently signed across contexts. The dataset is available in face-and-voice, face-only, and voice-only formats, but we only use the audio-video format. A single video may include more than one sentiment class. For example, a “positive” video may include periods of neutral sentiment. This study follows the original clip-level annotations provided in the RAVDESS dataset. Rather than manually segmenting videos during training, the entire video, including all frames, was assigned a single negative, neutral, or positive label to maintain consistency with the dataset annotations. This clip-level labeling introduces label noise when a clip contains segments of more than one underlying sentiment; its implications are discussed in Section 5.1.

The dataset was split 4:1 into training and validation sets. The training datasets for each of the four models (mouth, video only, audio only, and audio-video) were created separately using the same train-validation split. First, to create the mouth dataset, we prepared mouth images including open and closed samples from six actors in RAVDESS and an additional video from a web camera for one person. For each mouth, we ran the dlib landmark detector, extracted the mouth region, randomly sampled 832 mouth regions, and manually classified each sample mouth as open or closed. Second, to create the face dataset, we used 684 raw videos from RAVDESS, extracting series of face images from 18 actors into five categories: neutral, calm, happy, angry, and sad. Samples of these five emotions were placed into three categories: negative, neutral, and positive. Finally, the audio data comprised spectrogram images synchronized with the face images. We used pydub for audio segmentation, and the STFT was performed with Scipy.

The test data are organized into two categories: sampled complete video streams from RAVDESS and sampled video streams from a web camera. The RAVDESS data used for the final test set come from 6 actors (both male and female) not included in the training or validation sets. They include 192 RAVDESS video files. The distribution over the three categories was 48 positive, 48 neutral, and 96 negative. Second, we used 30 sampled video streams from web camera videos of 2 actors, providing an additional 5 negative, 20 neutral, and 5 positive videos. The distribution of training, validation, and test sets over the sentiment classes is shown in Table 1.

**Table 1:** Number of spectrogram and face images for each sentiment class in training, validation, and test sets, used in our study.

Database of emotional audio and video files (RAVDESS)	Positive (Happy)	Neutral (Calm and Neutral)	Negative (Angry and Sad)	Total
Training	96 Files Face image: 2,159 Spec image: 2,822	144 Files Face image: 3,222 Spec image: 4,083	190 Files Face image: 4,371 Spec image: 5,403	430 Files Face image: 9,752 Spec image: 12,308
Validation	32 Files Face image: 540 Spec image: 706	48 Files Face image: 806 Spec image: 1,021	64 Files Face image: 1,093 Spec image: 1,351	144 Files Face image: 2,439 Spec image: 3,078
Test	48 Files	48 Files	96 Files	192 Files

### 3.2 Experiment I: CNN

CNN networks were trained on 1) audio only, 2) only visual only, and 3) audio-visual data. The structure was the same as in Figures 4, 5, without the LSTMs. All three models used the Adam optimizer and default values of hyperparameters from previous work, with a batch size of 32 samples, a learning rate of 0.001, and categorical cross entropy loss. Each network was trained for 50 epochs. Training results are shown in Table 2.

**Table 2:** Training results.

Metric	CNN model			CNN-LSTM (Sequence length 10)			CNN-LSTM (Sequence length 50)
	A	V	AV	A	V	AV	AV
Image size	400 x 400	96 x 96	400 x 400 & 96 x 96	400 x 400	96 x 96	400 x 400 & 96 x 96	400 x 400 & 96 x 96
Training accuracy	43.00%	45.53%	87.23%	97.57%	63.75%	**100.00%**	57.97%
Training loss	1.04	1.05	0.34	0.21	0.86	**0.01**	0.97
Validation accuracy	45.00%	45.92%	78.55%	86.98%	63.04%	**91.30%**	51.09%
Validation loss	1.05	1.04	0.45	0.43	0.94	**0.33**	1.04

A: audio model, V: visual model, and AV: audio-visual model. Bold values indicate the final model (audio-visual CNN-LSTM, sequence length 10).

For the audio-only model, we obtained training accuracy, training loss, validation accuracy, and validation loss rates of 43.00%, 1.04, 45.00%, and 1.05, respectively. Training results for the visual only CNN model were similar, achieving 45.53% training accuracy and 45.92% validation accuracy. The comparable performance of the audio-only and visual-only models suggests that neither modality alone provided sufficient information for reliable sentiment classification, which is consistent with findings reported in Das and Singh (2023); Singh et al. (2024).

The audio-visual CNN model concatenates the audio CNN model and the visual CNN model as explained in Section 2.4, combined with the mouth CNN model from Section 2.2 for mouth classification. The audio-visual model achieved 87.23% training accuracy, 0.34 training loss, 78.55% validation accuracy, and 0.45 validation loss. Compared with the single modality models, the audio-visual model substantially improved both training and validation performance. This indicates that the integration of audio and visual information provides a more comprehensive understanding of human sentiments. The best accuracy in Experiment I overall was achieved with the audio-visual CNN model, which was far superior to both the audio-only CNN model and the visual-only CNN model. The audio-visual CNN was selected for the final CNN test. Test results are shown in column 3 of Table 3. The overall test accuracy was moderate, at 69.79%.

**Table 3:** Test results.

Class	Total number of video files	Experiment I audio-visual	Experiment II audio-visual	Alternative implementations audio-visual			
		CNN with MC	CNN-LSTM with MC	CNN-LSTM without MC	CNN-LSTM with MC version II	CNN-LSTM with MC	CNN-LSTM with MC version II
**Sequence length**		1	10	10	10	50	50
Positive	48 Files	34 Files	37 Files	**38 Files**	29 Files	32 Files	24 Files
Neutral	48 Files	22 Files	**37 Files**	36 Files	22 Files	32 Files	20 Files
Negative	96 Files	78 Files	**89 Files**	84 Files	89 Files	80 Files	88 Files
Total	192 Files	134 Files	**163 Files**	158 files	140 Files	144 Files	132 Files
Overall accuracy	100%	69.79%	**84.89%**	82.29%	72.91%	75%	68.75%

Number of correctly classified files for each condition is shown. MC: mouth classification. Bold values indicate the highest value in each row; in the Negative row, the proposed model ties with CNN-LSTM with MC version II.

### 3.3 Experiment II: CNN-LSTM

The CNN-LSTM models used a sequence length of 10 and were trained with stochastic gradient descent at a learning rate of 0.001, without momentum or weight decay. The audio-only and visual-only models were trained for 50 epochs, and the audio-visual model for 100 epochs with a batch size of 5. We began with the CNN of Experiment I and augmented each model by wrapping the entire sequence of CNN layers in a TimeDistributed layer with the number of LSTM layers as explained in Section 2. We used the same RAVDESS dataset for training and testing as in Experiment I. Separate CNN-LSTM networks were trained with audio only, visual only, and both audio and visual inputs.

Audio-only results (see Table 2) showed 97.57% training accuracy, 0.21 training loss, 86.98% validation accuracy, and 0.43 validation loss, representing a substantial improvement over the audio-only CNN model. The result indicates that temporal information captured by the LSTM improves sentiment recognition from audio signals.

The visual-only results were 63.75% training accuracy, 0.86 training loss, 63.04% validation accuracy, and 0.94 validation loss. Compared with the audio-only CNN-LSTM model, both training accuracy and validation accuracy were worse for the visual-only CNN-LSTM. Although the visual-only CNN-LSTM outperformed the visual-only CNN model, its performance remained considerably lower than the audio-only CNN-LSTM.

Finally, the audio-visual CNN-LSTM received both visual features (faces) and audio features (spectrograms). The model reached 100.00% training accuracy, 0.01 training loss, 91.30% validation accuracy, and 0.33 validation loss. The superior validation accuracy and lower validation loss demonstrate the benefit of combining multimodal information with temporal information. These training and validation results are superior to these of both the audio-only and visual-only models and are also superior to the best model from Experiment I. The learning curves of this model are shown in Figure 6. Training accuracy exceeds 99% from epoch 23 onward, whereas validation accuracy levels off after about 30 epochs, fluctuating between 82.61% and 92.39% (mean 88.23%) over epochs 31–100, and validation loss remains at approximately 0.34.

**Figure 6:**
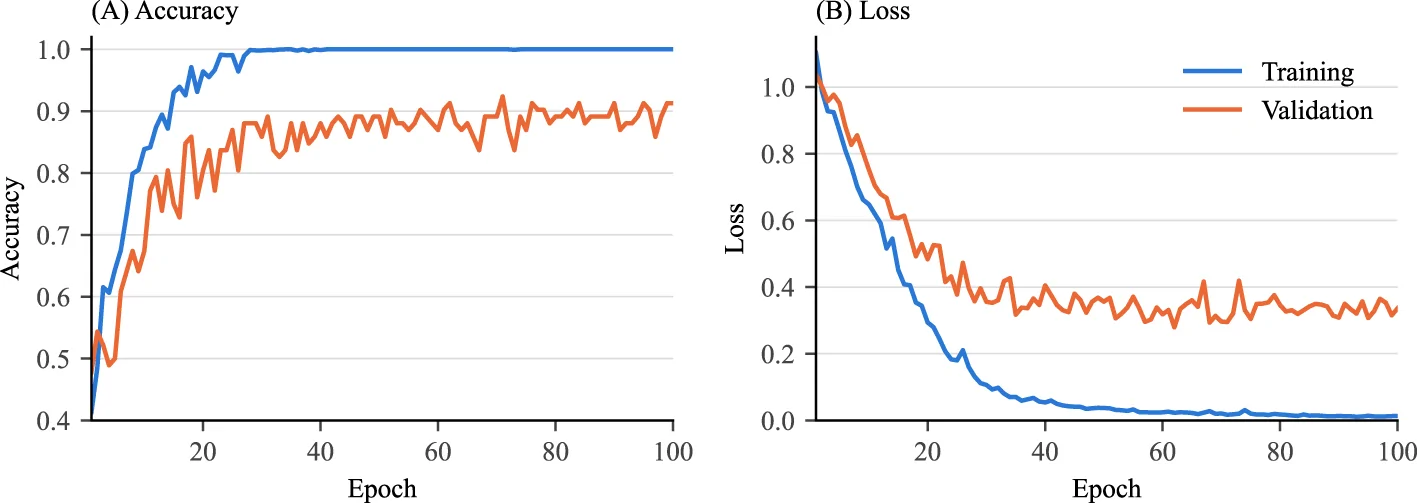
Training and validation accuracy **(A)** and loss **(B)** of the audio-visual CNN-LSTM over 100 training epochs.

On the test sets, the audio-visual CNN-LSTM model with mouth classification achieved an accuracy of 84.89% as shown in Table 3 (see also Figures 7, 8 for output examples generated by the model). Compared with the audio-visual CNN model from Experiment I (69.79%), the CNN-LSTM achieved an improvement of approximately 15 %. Therefore, the audio-visual CNN-LSTM with mouth classification was selected as the final model.

**Figure 7:**
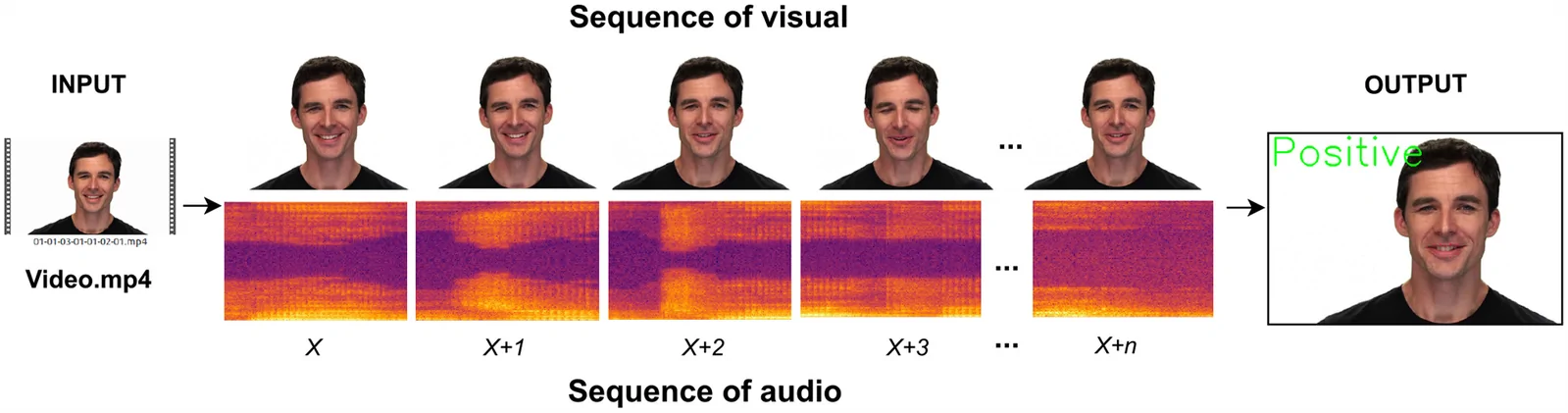
Output of sentiment analysis pipeline on RAVDESS video (input image adapted with permission from The Ryerson Audio-Visual Database of Emotional Speech and Song [RAVDESS], Livingstone and Russo, (2018), https://zenodo.org/records/1188976, licensed under CC BY-NC-SA 4.0).

**Figure 8:**
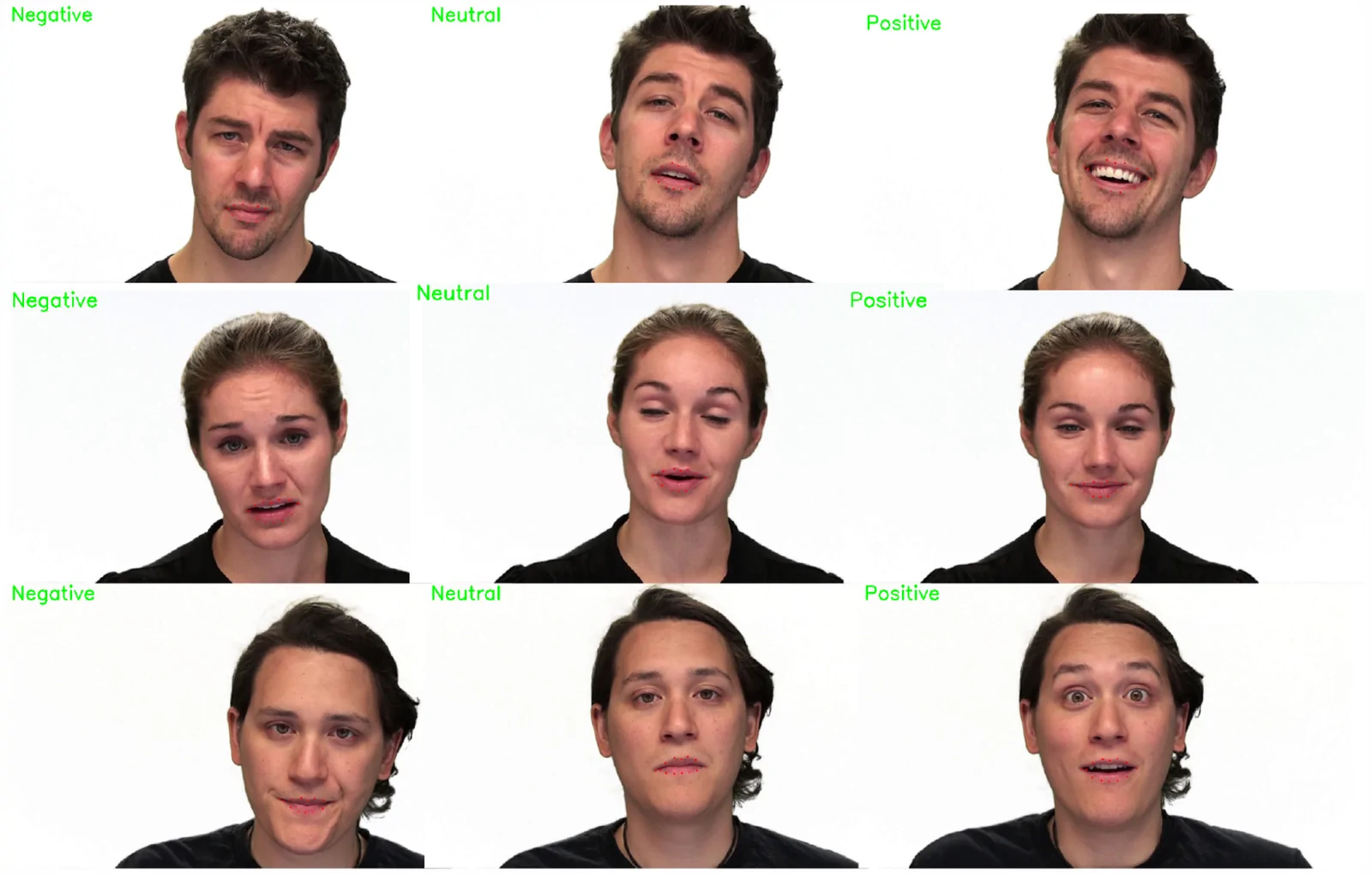
Outputs of the sentiment analyzer in three classes, i.e., negative, neutral, and positive, on RAVDESS video (input images adapted with permission from The Ryerson Audio-Visual Database of Emotional Speech and Song [RAVDESS], Livingstone and Russo, (2018), https://zenodo.org/records/1188976, licensed under CC BY-NC-SA 4.0).

Table 4 presents the accuracy results for the out-of-sample test set. The lower performance may be influenced by differences between the training and testing conditions. Specifically, RAVDESS contains professionally acted emotional expressions that are more exaggerated than natural daily expressions. Also, the RAVDESS actors are primarily Western participants, whereas the webcam evaluation involved Asian participants. This may affect model generalization. Additionally, positive sentiment recognition may be more difficult in real-world settings because people do not usually maintain exaggerated smiling expressions for extended periods. Additionally, the live evaluation was conducted using a Hikvision IR Cube Network Camera and Hikvision DS-7100 Series video recorder connected to a laptop-based inference system. During deployment, the proposed system processed live video streams continuously without observable interruption. Model inference latency is reported in Section 5.1.

**Table 4:** Accuracy of out-of-sample test set prediction.

Sentiment	Number of videos	Number of correct predictions (Audio-visual CNN-LSTM)
Negative	5	3
Neutral	10	9
Positive	5	0
Accuracy		60%

### 3.4 Alternative implementations

To test the importance of the mouth classification strategy, we consider an alternative design called *CNN-LSTM with MC version II* in which at time *t*, if the state of the mouth is predicted as open, then the sequence of face frames and corresponding spectrogram images are passed to the sequential audio-visual sentiment analysis model described in Section 2.4. When the mouth is predicted as closed at time *t*, the buffered sequence of faces is passed to the sequential visual sentiment analysis model described in Section 2.3.

As another alternative architecture, we test the importance of sequence length. We set the sequence length to either 10 or 50 and tested the model described in Section 2.2 as well as the CNN-LSTM with MC version II with both sequence lengths, obtaining four alternative methods. The audio-visual CNN-LSTM model with a sequence length 10 and no mouth classification obtained a prediction accuracy of 82.29%. The audio-visual CNN-LSTM with mouth classification version II and a sequence length of 10 achieved 72.91% accuracy. The audio-visual CNN-LSTM model with sequence length 50 obtained training accuracy of 57.97% and validation accuracy of 51.09%. The models with sequence length 50, with the mouth classification strategy of Section 2.2 or version II obtained test set accuracies of 75% and 68.75%, respectively. In summary, all of the alternative models were inferior to the proposed model, as shown in Table 3.

### 3.5 Experiment III: LLMs

A total of nine large language models (LLMs) were evaluated according to the validation set of the RAVDESS dataset in Experiment I. That is, it consists of 2,439 face images and 3,078 spectrogram images. These selected models cover a broad range of architectures and capabilities, including four large-scale general-purpose LLMs (gpt-4o, gemini-2.5 pro, llama4:scout, and deepseek-v2.5:236b), three vision-language models (qwen2.5vl:32b-q8_0, qwen2.5vl:72b, and llava:34b), and two mid-tier text-based LLMs (gemma3:27b-it-q8_0 and mistral-small3.2:24b-instruct-2506-q8_0).

Three sentiment analysis tasks were considered: (1) face images only, (2) spectrogram images only, and (3) a multimodal setting consisting of one face image and three corresponding spectrogram images. For Tasks (1) and (2), two prompting strategies were evaluated, namely zero-shot and three-shot prompting. For Task (3), only zero-shot prompting was employed to limit prompt complexity. All experiments were conducted under two decoding configurations: (i) temperature = 0 with top-p = 0.07 and (ii) temperature = 1 with top-p = 0.07. Each prompt was submitted three times, and majority voting was used to determine the final prediction. The prompt templates for all tasks are provided in Prompts 1–4. For the three-shot setting, three labeled examples corresponding to positive, neutral, and negative sentiment were included. Due to budget constraints, the experiments primarily focused on selected open-source LLMs, resulting in some incomplete evaluations for spectrogram and multimodal settings.

Prompt 1 **Face image.**

**System prompt:** You are an expert at analyzing facial expressions and body language to determine emotional sentiment.

<instructions>Focus on these visual cues:

- Facial expressions (smile, frown, eyebrow position, eye expression)
- Body posture and positioning
- Overall emotional cues visible in the image < /instructions>,

<output_format>Respond with exactly one word from these options: Positive, Negative, or Neutral. </output_format>,

**User prompt:** <task>Analyze the primary sentiment of the person in the provided image. </task>,

{*Image: Base64 format image*}

Prompt 2 **Face image with 3 shots.**

**System prompt:** You are an expert at analyzing facial expressions and body language to determine emotional sentiment.

<instructions>Identify an emotion visible in the provided face image. </instructions>,

<output_format>Respond with exactly one word from these options: Positive, Negative, or Neutral. </output_format>,

**3 Shot Examples:**

**User prompt:** <task>Analyze the primary sentiment in the provided image. </task>,

<image_description>Positive expression </image_description>,

{*Image: Base64 format image*}

**Assistant prompt:** <sentiment>Positive</sentiment>,

**User prompt:** <task>Analyze the primary sentiment in the provided image. </task>

<image_description>Negative expression </image_description>,

{*Image: Base64 format image*}

**Assistant prompt:** <sentiment>Negative </sentiment>,

**User prompt:** <task>Analyze the primary sentiment in the provided image. </task>

<image_description>Neutral expression </image_description>,

{*Image: Base64 format image*}

**Assistant prompt:** <sentiment>Neutral </sentiment>,

**User prompt:** <task>Analyze the primary sentiment in the provided image. </task>,

{*Image: Base64 format image*}

Prompt 3 **Spectrogram image.**

**System prompt:** You are an expert at analyzing spectrogram images to determine emotional sentiment.

<instructions>Identify an emotion visible in the provided spectrogram images. </instructions>,

<output_format>Respond with exactly one word from these options: Positive, Negative, or Neutral. </output_format>

**User prompt:** <task>Analyze the primary sentiment of the provided spectrogram image. </task>,

{*Image: Base64 format image*}

Prompt 4 **Face and Spectrogram images.**

**System prompt:** You are an expert at analyzing face and spectrogram images to determine emotional sentiment.

<instructions>Identify an emotion visible in the provided face and spectrogram images. For a face image, focus on these visual cues:

- Facial expressions (smile, frown, eyebrow position, eye expression)
- Body posture and positioning
- Overall emotional cues visible in the image </instructions>,

<output_format>Respond with exactly one word from these options: Positive, Negative, or Neutral. </output_format>,

**User prompt:** <task>Analyze the primary sentiment of the provided face and spectrogram images. </task>,

{*Face image: Base64 format image*}

{*Spectrogram image 1: Base64 format image*}

{*Spectrogram image 2: Base64 format image*}

{*Spectrogram image 3: Base64 format image*}

The results of the face image experiments are summarized in Table 5. Overall, both zero-shot and three-shot prompting yielded comparable performance across most models. Indeed, gemini-2.5-pro achieved the highest accuracy of 69.9% under the temperature-0 configuration, while gemma3:27b-it-q8_0 attained the highest accuracy under the temperature-1 setting, reaching 70.8% in the zero-shot case and 65.5% in the three-shot case. With the exception of deepseek-v2.5:236b, the remaining models achieved accuracies ranging from 56.0% to 70.8%, with corresponding F1-scores between 45.9% and 70.6%, as in Table 6.

**Table 5:** Accuracy of 9 LLM models.

Accuracy of models	Face 0 shot		Face 3 shot		Spectrogram 0 shot		Spectrogram 3 shot		Face and Spectrogram 0 shot	
	T 0, P 0.07	T 1, P 0.07	T 0, P 0.07	T 1, P 0.07	T 0, P 0.07	T 1, P 0.07	T 0, P 0.07	T 1, P 0.07	T 0, P 0.07	T 1, P 0.07
1. gpt-4o	0.646	–	0.663	–	–	–	–	–	–	–
2. gemini-2.5-pro	**0.699**	–	**0.699**	–	–	–	–	–	–	–
3. gemma3:27b-it-q8_0	0.696	**0.708**	0.655	**0.655**	0.435	0.435	0.311	0.311	0.137	0.209
4. mistral-small3.2:24b-instruct-2506-q8_0	0.656	0.695	0.635	0.635	0.379	0.379	**0.352**	**0.352**	0.221	0.221
5. llama4:scout	0.620	0.616	0.565	0.568	0.336	0.336	0.326	0.328	0.221	0.221
6. qwen2.5vl:32b-q8_0	0.564	0.597	0.579	0.579	0.331	0.331	0.317	0.314	0.330	0.330
7. qwen2.5vl:72b	0.651	0.647	0.560	0.560	0.338	0.338	0.324	0.324	0.330	0.330
8. llava:34b	0.696	0.696	0.590	0.590	0.437	0.437	0.258	0.258	**0.448**	**0.448**
9. deepseek-v2.5: 236b	0.121	0.163	0.221	0.221	**0.439**	**0.439**	0.332	0.332	0.330	0.330

–: not evaluated. Owing to API cost, the two commercial models (gpt-4o and gemini-2.5-pro) were evaluated only on face images at temperature 0. Bold values indicate the highest accuracy in each column.

**Table 6:** F1 score of 9 LLM models.

F1 score of models	Face 0 shot		Face 3 shot		Spectrogram 0 shot		Spectrogram 3 shot		Face and Spectrogram0 shot	
	T 0, P 0.07	T 1, P 0.07	T 0, P 0.07	T 1, P 0.07	T 0, P 0.07	T 1, P 0.07	T 0, P 0.07	T 1, P 0.07	T 0, P 0.07	T 1, P 0.07
1. gpt-4o	0.664	–	0.677	–	–	–	–	–	–	–
2. gemini-2.5-pro	0.683	–	**0.683**	–	–	–	–	–	–	–
3. gemma3:27b-it-q8_0	**0.700**	**0.706**	0.658	**0.658**	**0.322**	**0.322**	0.296	0.296	0.080	0.115
4. mistral-small3.2:24b-instruct-2506-q8_0	0.669	0.700	0.639	0.639	0.258	0.258	0.318	0.318	0.121	0.121
5. llama4:scout	0.633	0.625	0.563	0.563	0.321	0.321	**0.325**	**0.327**	0.121	0.121
6. qwen2.5vl:32b-q8_0	0.573	0.612	0.586	0.586	0.169	0.169	0.249	0.247	0.166	0.166
7. qwen2.5vl:72b	0.667	0.664	0.555	0.555	0.227	0.227	0.273	0.273	0.166	0.166
8. llava:34b	0.682	0.682	0.459	0.459	0.262	0.262	0.206	0.206	**0.206**	**0.206**
9. deepseek-v2.5: 236b	0.072	0.094	0.121	0.121	0.203	0.203	0.166	0.166	0.166	0.166

–: not evaluated. Owing to API cost, the two commercial models (gpt-4o and gemini-2.5-pro) were evaluated only on face images at temperature 0. Bold values indicate the highest F1 score in each column.

In contrast, performance on spectrogram-only sentiment analysis was substantially lower across all models. Interestingly, deepseek-v2.5:236b, which performed poorly in the face image task, achieved the highest accuracy in the spectrogram zero-shot setting, reaching 43.9%. The second-best performance was obtained by mistral-small3.2:24b-instruct in the three-shot configuration, with an accuracy of 35.2%. These results were consistent across both decoding configurations, meaning that current LLMs may have limited capability to interpret spectrogram representations for sentiment inference.

For the multimodal audio-visual task, overall performance remained limited. Indeed, llava:34b achieved the highest accuracy of 44.8% under both decoding configurations, while the remaining models obtained accuracies ranging from 13.7% to 33.0%. These findings suggest that, despite their multimodal capabilities, current LLM-based systems face significant challenges when jointly reasoning over synchronized audio and visual inputs for sentiment analysis.

In summary, the experimental results reveal several important observations. (1) Different LLMs exhibit task-dependent performance, with no single model consistently outperforming others across all input modalities. (2) Spectrogram-based sentiment analysis remains particularly challenging for current LLMs, indicating limited capability in interpreting low-level acoustic representations. (3) Although multimodal LLMs demonstrate some ability to process combined audio-visual inputs, their performance remains substantially below that of specialized deep learning architectures. (4) For face-only sentiment classification, gemma3:27b (70.8%) and Gemini-2.5-pro (69.9%) outperform the CNN (45.92%) and CNN-LSTM (63.04%) baselines. However, when both audio and visual modalities are jointly considered, the proposed CNN-LSTM framework achieves a significantly higher accuracy of 91.30%, whereas all evaluated LLMs remain below 50%. These results highlight the continued effectiveness of lightweight, task-specific multimodal models for real-time sentiment analysis and underscore current limitations of general-purpose LLMs in this domain.

## 4 Comparison with state of the art

A comparison of the proposed model and prior work is summarized in Table 7. Most RAVDESS studies address the eight-class emotion recognition task, whereas the present work addresses the coarser three-class sentiment task; the two are not directly comparable, and Table 7 therefore reports emotion classification and sentiment analysis accuracy in separate columns. Within this setting, our approach attains competitive accuracy with a substantially smaller parameter count than the reproduced audio-visual baselines. Most of the existing work using the RAVDESS dataset focuses on speech emotion recognition (SER). Kwon et al. propose a deep stride convolutional neural network (DSCNN) architecture for speech emotion recognition (Kwon et al., 2020). They transform audio data to spectrograms as in the current work. Their method achieves accuracy up to 79.5%. Although our work is not focused on speech emotion recognition, to compare performance, we created a new model based on their proposed architecture then trained and evaluated it on the same dataset used for evaluation in Section **??**. Despite differences in experimental settings and task definitions, our model demonstrated competitive performance.

**Table 7:** Prior work on RAVDESS using face and/or speech data.

Paper	Feature extraction technique	Classifiers	Reported emotion classification accuracy	Sentiment analysis accuracy
Parry et al. (2019)	Mel filterbank coefficients	LSTM, CNN, and CNN-LSTM	-	53.97%, 65.67% and 53.08%
Kwon et al. (2020)	Spectrogram	DSCNN	79.50%	35%
Lin et al. (2020)	Face images	CNN (AlexNet) and CNN-LSTM	43.17% and 74.07%	61%
Song et al. (2021)	Face images and spectrogram	VGG16 -1GRU with VGG16-3GRU	90%	66.66%
Yakaew et al. (2021)	Face images and spectrogram	CNN	-	69.79%
Mocanu et al. (2023)	Face images and spectrogram	3D ResNet-101 and ResNet-18, cross-attention fusion	89.25%	-
Sun et al. (2024)	Face images and spectrogram	Transformer encoders, cross-modal fusion encoder	87.96%	-
Fan et al. (2024)	Pre-trained audio and visual features (non-end-to-end)	Attention aggregation fusion	88.00%	-
Zhang et al. (2025)	Face images and MFCCs	CNN and EfficientFace, cross-modal attention fusion	88.67%	-
**Our approach**	Face images and spectrogram	CNN-LSTM	-	**84.89%**

Sentiment analysis accuracy refers to the three-class task (negative/neutral/positive) addressed in this work; emotion classification accuracy is reported for each paper's original task (e.g., eight emotions) and is not directly comparable. Bold values indicate the proposed approach.

In Parry et al. (2019)'s work on speech emotion recognition using speech information from RAVDESS, they propose three models: CNN, LSTM, and CNN-LSTM. Their goal was the same as ours i.e. classifying sentiment into three classes: negative, neutral and positive. All three models were single networks without mouth classification. The three models were evaluated on RAVDESS. Test accuracy was 65.67% for the CNN, 53.97% for the LSTM, and 53.08% for the CNN-LSTM. Both the CNN and LSTM models achieved higher performance than the CNN-LSTM model, conflicting with our results. While direct comparison may be limited by differences in experimental design, our audio-visual CNN-LSTM model outperforms both of the models of Parry et al. (2019).

Among work that focuses on visual data for emotion recognition from RAVDESS, Lin et al. (2020) present a CNN-LSTM model for recognizing facial emotion and two levels of sentiment. This work does not use a detector or run in real time. They apply AlexNet over the entire image and add a LSTM. Recognition accuracy was 74.07% over the five emotions, and 84.33% over the positive and negative sentiments. We created a model following the architecture of Lin et al. (2020) for sentiment analysis and compared with our system. The new model obtained a validation accuracy of 48.91%, and the test accuracy was 61%, lower than that of our system. The model of Lin et al. (2020) uses a single network and does not integrate any additional techniques. This straightforward approach is apparently inferior to the fused audio-visual CNN-LSTM with mouth classification described here.

Yakaew et al. (2021) present a fusion method using audio features and visual features with CNNs only. They manually segmented the RAVDESS video data samples into segments containing very consistent negative, neutral, and positive sentiments. In contrast, our work uses all frames from each video for training the audio-only CNN, visual-only CNN, and audio-visual CNN, with a single label for each entire video. Additionally, while the previous system used frame-by-frame analysis to select a visual or audio-visual network, our proposed approach incorporates mouth classification over 10-frame sequence to capture temporal visual information and improve performance. The results here show that the audio-visual CNN model obtains higher accuracy than the audio CNN or visual CNN models. The evaluation settings also differ between the two studies. Yakaew et al. (2021) used a relatively small test set consisting of 54 files (18 negative, 18 neutral, and 18 positive), while our proposed work increased the number of test files to 192. The audio-visual CNN from Yakaew et al. (2021) achieved 69.79% accuracy, whereas the current work achieved 84.89% prediction accuracy on the same dataset.

Song et al. (2021) propose an audio-visual emotion recognition system for classifying video into eight emotions. Their architecture is similar to ours, with parallel visual (VGG16-GRU) and audio (VGG16-3GRU) CNNs. Both audio features and visual features are used for training and testing. The model comprises more than 270 million parameters in total, much more than our model. The audio and visual networks were fused to achieve 90% accuracy on a RAVDESS dataset. We created a new model following their structure and found that our model is more accurate than the model of Song et al. (2021) on the same dataset, despite differences in task objectives.

Recent audio-visual systems evaluated on RAVDESS report accuracies of approximately 87–89% on the eight-class emotion recognition task, using cross-modal attention or self-supervised pre-training (Mocanu et al., 2023; Sun et al., 2024; Fan et al., 2024; Zhang et al., 2025). Earlier audio-visual work reported 86.70% under speaker-independent 5-fold cross-validation (Luna-Jiménez et al., 2022), and an audio-only model has been reported to reach 91.9% on seven of the eight emotion classes with a stratified 80:20 split (Waleed and Shaker, 2025). Because these studies address a finer-grained emotion task, and because reported results vary considerably with dataset splits, preprocessing, and evaluation protocols (Kumar et al., 2025), they are not directly comparable with our three-class sentiment result; the four audio-visual systems above are therefore listed in Table 7 with their reported emotion classification accuracy only. Three-class valence labeling is nevertheless an established formulation in affective computing, used for example in the FAU Aibo and SEED corpora (Kumar et al., 2025). In terms of model size, our network (20.9M parameters) is an order of magnitude smaller than the audio-visual system of Song et al. (2021) (more than 270M parameters), although audio-only models can be smaller still (e.g., 1.2M parameters in Waleed and Shaker, 2025) at the cost of discarding visual cues.

## 5 Discussion and conclusion

This paper presented a multimodal audio-visual neural network designed for low-latency, frame-by-frame sentiment prediction from video streams. The proposed framework incorporates a dynamic mouth state classification mechanism that selectively activates processing pathways depending on whether the mouth is open or closed, enabling more effective exploitation of speech-related visual cues. The model was evaluated on the RAVDESS dataset, where the CNN-LSTM architecture achieved a test accuracy of 84.89% when jointly modeling audio and visual information, outperforming unimodal baselines. Ablation studies further demonstrated that a short temporal context (10 frames, approximately 350 ms) yields higher accuracy than longer sequences (50 frames, approximately 1750 ms), primarily because shorter windows generate a larger number of training sequences and allow the model to better capture local affective dynamics. The aggregated mouth-based processing strategy was shown to improve recognition performance for neutral and negative sentiment categories, although a slight degradation was observed for positive sentiment.

A small out-of-sample webcam evaluation (20 videos from two actors) was conducted as a preliminary feasibility check; its size does not support conclusions about generalization. This evaluation revealed reduced performance for positive sentiment, including 0 of 5 correct predictions on positive-sentiment clips, while performance on neutral and negative classes was relatively stronger. We attribute part of this gap to the controlled recording conditions of RAVDESS, including uniform head pose, stable illumination, exaggerated emotional expressions, and minimal background noise, versus the greater variability in facial motion, background complexity, and acoustic conditions in the webcam recordings. We also note that RAVDESS actors are Western professionals instructed to produce sustained, exaggerated expressions on cue, whereas the webcam subjects were interacting naturally; posed positive expressions of this kind tend to be more prolonged and exaggerated than spontaneous ones (Elfenbein and Ambady, 2002), which may partly explain why the positive class transferred least well. Improving the recognition of spontaneous positive expressions, and evaluating the system on a substantially larger and more diverse out-of-sample set, remain important directions for future work.

Beyond conventional deep learning models, this work also investigated the applicability of large language models (LLMs) for sentiment analysis using visual, audio-derived spectrogram, and multimodal inputs. The LLM-based experiments demonstrated that, although some models achieve competitive performance on face-only sentiment classification, their effectiveness substantially degrades when applied to spectrogram-based or combined audio-visual inputs. In particular, all evaluated LLMs achieved less than 50% accuracy in the multimodal setting, whereas the proposed CNN-LSTM framework attained significantly higher accuracy. These results suggest that, despite recent advances in multimodal LLMs, general-purpose models remain limited in their ability to reason over synchronized low-level audio-visual representations for real-time sentiment analysis.

### 5.1 Limitations

This study has several limitations that should be considered when interpreting the reported results.

#### 5.1.1 Evaluation protocol

Results are reported from a single 4:1 train/validation split with a held-out six-actor test set, rather than speaker-independent *k*-fold cross-validation with mean and standard deviation across folds, which is the more common protocol in recent RAVDESS literature. The validation set was drawn at random at the clip level, so clips from the same actors appear in both the training and validation sets; the validation accuracy (91.30%) is therefore likely optimistic, and the speaker-independent six-actor test set (84.89%) provides the more reliable estimate of generalization. A single split does not characterize the variance of the reported accuracy, and 84.89% should be read as a point estimate rather than a variance-characterized one. Cross-validation was not performed in the current study owing to resource constraints. To support independent verification and future cross-validation studies, the prepared face and spectrogram dataset used in this work is publicly available (see Data Availability Statement). We identify speaker-independent 5-fold cross-validation, using this released data, as a priority for follow-up work.

#### 5.1.2 Runtime profiling

The system was deployed and observed to process live video streams without observable interruption using a Hikvision IR Cube Network Camera and a laptop-based inference pipeline. Model inference latency was subsequently measured for the trained networks (the audio-visual CNN-LSTM and the mouth-state CNN, 20.9M parameters in total) on an Apple M4 Max laptop (16-core CPU, 40-core GPU, 128 GB unified memory; TensorFlow 2.16 with Metal acceleration). For one 10-frame sequence at batch size 1, the median latency of the audio-visual branch, including mouth-state classification of the ten frames, was 34.4 ms (95th percentile 35.0 ms; approximately 29 sequences per second) on the GPU and approximately 0.34–0.44 s on the CPU alone, with a peak resident memory of about 2.2 GiB in the CPU configuration. These figures cover model inference only; face detection, mouth cropping, spectrogram computation, and video and audio input/output were not included, and the hardware differs from the laptop used in the original deployment. GPU inference time is therefore comparable to the 35 ms frame interval, but end-to-end real-time throughput of the complete pipeline was not established; profiling the full pipeline on representative deployment hardware is left for future work.

#### 5.1.3 Overfitting

The final audio-visual CNN-LSTM model reached 100.00% training accuracy versus 91.30% validation accuracy. Regularization in the current architecture is limited to 20% dropout applied to the LSTM layers, with a fixed training schedule of 100 epochs and no early stopping. The learning curves (Figure 6) show training accuracy saturating after about 23 epochs while validation loss stops decreasing after about 30 epochs, a pattern consistent with overfitting. We identify early stopping, weight decay, and data augmentation as concrete steps to reduce this gap in future work.

#### 5.1.4 Label noise

As noted in Section 2, a single clip-level label was assigned to each video even though a clip may contain segments expressing more than one sentiment (e.g., a brief neutral segment within a predominantly positive clip). We did not quantify the resulting label noise or its effect on the reported metrics in this study; frame- or segment-level relabeling is a natural direction for future work and may be particularly relevant to the neutral/negative confusion patterns observed in our results.

### 5.2 Future work

Future work will focus on improving multimodal fusion strategies, expanding the diversity and realism of training data, and investigating hybrid architectures that combine the reasoning capabilities of LLMs with the efficiency and inductive biases of task-specific neural models. Beyond the specific items noted above, we also plan to explore lightweight cross-modal attention in place of feature concatenation, soft (probability-weighted) mouth-state gating in place of the current hard threshold, and class-weighted or focal-loss training to address the class imbalance in the test set, together with macro-F1 reporting. We further plan to evaluate cross-corpus generalization on other audio-visual emotion datasets and to provide audio to LLMs as waveform features or speech transcripts rather than spectrogram images. It is hoped that the findings of this study, together with the released dataset, will stimulate further research into robust, real-time multimodal sentiment analysis systems suitable for practical deployment.

## Data Availability

The datasets presented in this study can be found in online repositories. The names of the repository/repositories and accession number(s) can be found below: doi.org/10.5281/zenodo.23227107.

## Footnotes

1 The face images of RAVDESS actors shown in Figures 2, 4, 5, 7, 8 are reproduced from RAVDESS (Livingstone and Russo, 2018), licensed under CC BY-NC-SA 4.0 (https://creativecommons.org/licenses/by-nc-sa/4.0/).
